# Lymph node ratio predicts adjuvant chemotherapy benefit in esophageal squamous cell carcinoma

**DOI:** 10.1093/oncolo/oyaf315

**Published:** 2025-09-25

**Authors:** Jun Peng, Yi Wang, Haoyue Hu, Lei Wu, Wei Dai, Yehan Zhou, Na Li, Lin Peng, Xuefeng Leng, Xiang Zhuang, Qifeng Wang, Xiang Wang

**Affiliations:** Department of Thoracic Surgery, Sichuan Clinical Research Center for Cancer, Sichuan Cancer Center, Sichuan Cancer Hospital & Institute, University of Electronic Science and Technology of China, Chengdu, 610041, China; Department of Radiation Oncology, Radiation Oncology Key Laboratory of Sichuan Province, Sichuan Clinical Research Center for Cancer, Sichuan Cancer Center, Sichuan Cancer Hospital & Institute, University of Electronic Science and Technology of China, Chengdu, 610041, China; Department of Radiation Oncology, Sun Yat-sen University Cancer Center, State Key Laboratory of Oncology in South China, Collaborative Innovation Center for Cancer Medicine, Sun Yat-sen University, Guangzhou, Guangdong, 510060, China; Department of Radiation Oncology, Radiation Oncology Key Laboratory of Sichuan Province, Sichuan Clinical Research Center for Cancer, Sichuan Cancer Center, Sichuan Cancer Hospital & Institute, University of Electronic Science and Technology of China, Chengdu, 610041, China; Department of Thoracic Surgery, Sichuan Clinical Research Center for Cancer, Sichuan Cancer Center, Sichuan Cancer Hospital & Institute, University of Electronic Science and Technology of China, Chengdu, 610041, China; Department of Pathology, Sichuan Clinical Research Center for Cancer, Sichuan Cancer Hospital & Institute, Sichuan Cancer Center, University of Electronic Science and Technology of China, Chengdu, 610041, China; Cancer Center of Suning Central Hospital, Suining, 629000, China; Department of Thoracic Surgery, Sichuan Clinical Research Center for Cancer, Sichuan Cancer Center, Sichuan Cancer Hospital & Institute, University of Electronic Science and Technology of China, Chengdu, 610041, China; Department of Thoracic Surgery, Sichuan Clinical Research Center for Cancer, Sichuan Cancer Center, Sichuan Cancer Hospital & Institute, University of Electronic Science and Technology of China, Chengdu, 610041, China; Department of Thoracic Surgery, Sichuan Clinical Research Center for Cancer, Sichuan Cancer Center, Sichuan Cancer Hospital & Institute, University of Electronic Science and Technology of China, Chengdu, 610041, China; Department of Radiation Oncology, Radiation Oncology Key Laboratory of Sichuan Province, Sichuan Clinical Research Center for Cancer, Sichuan Cancer Center, Sichuan Cancer Hospital & Institute, University of Electronic Science and Technology of China, Chengdu, 610041, China; Department of Thoracic Surgery, Sichuan Clinical Research Center for Cancer, Sichuan Cancer Center, Sichuan Cancer Hospital & Institute, University of Electronic Science and Technology of China, Chengdu, 610041, China

**Keywords:** esophageal squamous cell carcinoma, lymph node ratio, adjuvant chemotherapy, restricted cubic splines, propensity score matching

## Abstract

**Background:**

The lymph node ratio (LNR) has emerged as an important prognostic factor in various cancers, including esophageal squamous cell carcinoma (ESCC). This study aimed to evaluate the utility of LNR in guiding decisions for adjuvant chemotherapy in ESCC patients following resection.

**Materials and Methods:**

A retrospective analysis was conducted on 2267 patients who underwent potentially curative surgery for ESCC at Sichuan Cancer Hospital and Institute between January 2010 and December 2017. Univariate and multivariate Cox proportional hazards regressions were used to assess factors influencing overall survival (OS), with LNR analyzed using restricted cubic splines (RCS) to explore its relationship with treatment and survival outcomes. Propensity score matching (PSM) was employed to adjust for biases between treatment groups.

**Results:**

Among the patients, 1416 underwent surgery alone (S group) and 851 received surgery plus adjuvant chemotherapy (S + CT group). The median LNR was 3%, with an interquartile range of 0%-12%. RCS analysis identified an LNR threshold of 11%, below which patients showed a significant OS benefit from adjuvant chemotherapy (hazard ratio [HR]: 0.57; 95% CI: 0.46-0.71; *P* < 0.001). However, patients with an LNR above 11% did not derive any OS benefit from chemotherapy (HR: 0.87; 95% CI: 0.70-1.09; *P* = 0.238).

**Conclusion:**

These findings suggest that LNR is a valuable marker for identifying ESCC patients who would benefit from postoperative adjuvant chemotherapy. A threshold LNR of 11% can help personalize treatment strategies, and patients with higher LNRs may require more intensive approaches like chemoradiotherapy to improve survival. Further prospective studies are needed to validate these results.

Implications for Practice Postoperative adjuvant chemotherapy is often considered ineffective for most esophageal squamous cell carcinoma (ESCC) patients. This study used restricted cubic spline (RCS) analysis to explore the role of lymph node ratio (LNR) in predicting adjuvant chemotherapy benefits. The results showed that adjuvant chemotherapy improved survival for patients with an LNR below 11%, while it provided no benefit for those with higher LNRs. An LNR threshold of 11% can personalize treatment, identifying those who may need chemotherapy or more intensive treatments like chemoradiotherapy. These findings could refine postoperative ESCC treatment, though further studies are needed to validate them.

## Introduction

Neoadjuvant chemoradiotherapy has emerged as the standard treatment for patients with resectable locally advanced esophageal squamous cell carcinoma (ESCC), according to the CROSS and Chinese 5010 trials.^1^^,^^2^ Currently, surgical resection without neoadjuvant therapy is recommended exclusively for patients clinically staged as cT1b-cT2N0. However, in clinical practice, a subset of patients do not undergo neoadjuvant chemoradiotherapy due to factors including resource limitations at non-specialized institutions, concerns about increased post-operative morbidity associated with preoperative therapy, and the inherent inaccuracies in clinical staging. For patients undergoing surgery alone, the prognosis is poor, with a 5-year survival rate of less than 50%.^2^^,^^3^ Furthermore, according to the National Comprehensive Cancer Network (NCCN) guidelines, adjuvant therapy is not recommended for patients with ESCC following an R0 resection, unlike the approach for esophageal adenocarcinoma.^4^ However, the guidelines of the Japan Esophageal Society (JES) continue to recommend surgery followed by adjuvant chemotherapy as an alternative treatment option.^5^ This ongoing controversy is partly due to the lack of evidence from large-scale phase III clinical trials, and partly due to the debated efficacy of postoperative treatment, including adjuvant chemotherapy and radiotherapy.^6–11^ A major issue is the failure to select appropriate patient populations for these treatments.

The LNR—the ratio of pathologically positive lymph nodes (LN) to all nodes examined—has emerged as a potential prognostic indicator in various cancers, including ESCC.^12–16^ LNR accounts for both the total number of LN examined and the number of positive nodes, thereby partially reflecting tumor burden. This study aims to validate LNR’s prognostic value in resected ESCC patients and evaluate its ability to identify those who may benefit from adjuvant chemotherapy.

## Materials and methods

### Patient selection

This retrospective study utilized a database of esophageal cancer from the Sichuan Cancer Hospital and Institute, covering the period from January 2010 to December 2017. This study was approved by the ethics committee of our institution (ethical approval number SCCHEC-02–2023-029). The inclusion criteria were: esophagectomy performed at our hospital, a pathological diagnosis of ESCC, and tumor location in the thoracic region. The exclusion criteria included: any perioperative antitumor treatment, adjuvant chemoradiotherapy or radiotherapy post-esophagectomy, absence of resected LN during surgery, postoperative 90-day mortality, R1 or R2 esophagectomy, pathological T stage Tis/unknown or M1 stage, and missing required data (Figure S1). Patient demographics (sex and age), tumor location, surgical details (type of procedure, number of nodes examined), pathological details (tumor grade, tumor, node and metastasis (TNM) stage, angioinvasion, perineural invasion, tumor size, and number of positive nodes), treatment (surgery alone or surgery followed by adjuvant chemotherapy), and outcome variables (survival status and follow-up period) were collected.

### Surgery and adjuvant chemotherapy

Esophagectomy was primarily performed using the right transthoracic procedure (Ivor-Lewis or McKeown). The surgical approach was determined by the tumor’s location and the surgeon’s preference. Disease staging was classified according to the American Joint Committee on Cancer’s 8th edition of the TNM system. There are no standardized protocols for treating ESCC patients postoperatively. Consequently, decisions on adjuvant chemotherapy were made based on the patient’s preferences or financial circumstances, hospital policies, the doctor’s judgment, and tumor stage. Adjuvant chemotherapy usually consists of 4-6 cycles of platinum-based medications, with or without 5-fluorouracil or paclitaxel (Supplementary Table S1), and the dosage varies per patient. In this study, adjuvant chemotherapy was defined as receiving at least 2 cycles of treatment. Patients were followed up every 3 months for the first 2 years; thereafter, follow-ups occurred every 6 months for 3-5 years.

### Statistical analyses

Clinical variables were presented as whole numbers and percentages, while continuous variables were summarized using medians with interquartile ranges (IQRs) or means with standard deviations (SDs), depending on their distribution. Initially, the LNR was analyzed as a continuous variable. Univariate and multivariate Cox proportional hazards regressions were performed to assess the impact of clinical variables on overall survival (OS). Factors with a *P* < 0.1 in univariate analysis were included in the multivariate Cox regression model. Spearman’s correlation coefficient was used to analyze the correlation between LNR, the number of examined LN, the number of positive LN, and the pathological *N* stage. Strongly correlated factors with LNR were excluded from the multivariate Cox regression analysis.

Additionally, restricted cubic splines (RCSs) were utilized to evaluate the relationships between LNR and OS, and between LNR, treatment, and OS on a continuous scale. RCS were used to identify inflection points (ie, thresholds) in the risk function. The RCS analysis was based on a multivariable Cox model adjusted for factors significantly associated with OS, with 3 knots set at the 10th, 50th, and 90th percentiles of LNR. After determining the LNR threshold through RCS, unpaired *t*-tests were used for normally distributed variables, while Mann-Whitney *U* tests or Kruskal-Wallis tests were used for non-normally distributed variables to assess baseline levels in subgroups. Propensity score matching (PSM) with a 1:1 ratio and 0.05 caliper was used for subgroup bias adjustment. OS was calculated from the date of surgery using Kaplan-Meier estimates, and the log-rank test was employed to assess the equality of survival functions. Statistical analyses were performed using R (v4.0.4), with *P* < 0.05 considered indicative of statistical significance.

## Results

### Patient characteristics

A total of 2267 consecutive ESCC patients (median age, 62 years; IQR: 57-67 years) who underwent potentially curative resection at our institution between January 2010 and December 2017 were examined. In the overall cohort, the median number of nodes examined and nodes positive was 20 (IQR: 14-27) and 1 (IQR: 0-2), respectively, with a median LNR of 3% (IQR: 0-12). A total of 1416 patients (62%) received surgery alone (S group), while 851 (38%) received surgery followed by chemotherapy (S + CT group). The clinical and pathological data of the patients are summarized in Table 1.

**Table 1.** Patient characteristics.

Variable	***N*** **=** **2267** ^a^ (%)
**Sex**	
** Female**	430 (19)
** Male**	1837 (81)
**Age (median [IQR])**	62 (57, 67)
**Tumor location**	
** Upper third**	532 (23)
** Middle third**	1219 (54)
** Lower third**	516 (23)
**Tumor grade**	
** G1**	372 (16)
** G2**	922 (41)
** G3**	872 (38)
** Unknown**	101 (4.5)
**Procedure type**	
** Ivor-lewis**	626 (28)
** Mckeown**	1600 (71)
** Others**	41 (1.8)
**Pathologic *T* stage**	
** T1**	279 (12)
** T2**	459 (20)
** T3**	1372 (61)
** T4**	157 (6.9)
**Pathologic *N* stage**	
** N0**	1084 (48)
** N1**	634 (28)
** N2**	366 (16)
** N3**	183 (8.1)
**Angioinvasion**	
** No/unknown**	1894 (84)
** Yes**	373 (16)
**Perineural invasion**	
** No/unknown**	1857 (82)
** Yes**	410 (18)
**Tumor size (median [IQR])**	40 (26, 50)
**Number of nodes examined (median [IQR])**	20 (14, 27)
**Number of nodes positive (median [IQR])**	1.00 (0.00, 2.00)
**Lymph node ratio (median [IQR])**	3 (0, 12)
**Treatment**	
** S group**	1416 (62)
** S + CT group**	851 (38)

a *n* (%); Median (IQR).

Abbreviations: CT: chemotherapy; IQR: interquartile ranges; S: surgery.

### Univariate and multivariate analyses for OS

During a median follow-up period of 38 months (range, 2-115 months), the median OS was 50.8 months (95% confidence interval [CI]: 45.6-57 months), with a 5-year survival rate of 46.1%. In univariate analysis, sex, tumor grade, procedure type, pathologic T stage, pathologic N stage, angioinvasion, perineural invasion, tumor size, number of positive nodes, and LNR were found to be significant prognostic factors (Table 2). Moreover, LNR was significantly related to the number of positive nodes (*R* = 0.970; *P* < 0.001) and pathologic N stage (*R* = 0.964; *P* < 0.001; Figure S2). Therefore, we removed the number of positive nodes and pathologic *N* stage from the multivariate analysis. In multivariate analysis, sex, tumor grade, pathologic *T* stage, angioinvasion, perineural invasion, tumor size, LNR, and treatment were found to be independent prognostic factors for OS (Table 2).

**Table 2.** Univariate and multivariate Cox model for overall survival in the whole cohort.

	Univariate analysis			Multivariate analysis		
Characteristic	**HR**	95% CI	*P*	HR	95% CI	*P*
**Sex**						
** Female**	—	—		—	—	
** Male**	1.70	1.43, 2.03	<0.001	1.56	1.30, 1.86	<.001
**Age**	1.01	1.00, 1.01	0.133			
**Tumor location**						
** Upper third**	—	—				
** Middle third**	0.97	0.84, 1.13	0.720			
** Lower third**	0.98	0.82, 1.17	0.836			
**Tumor grade**						
** G1**	—	—		—	—	
** G2**	1.43	1.18, 1.73	<0.001	1.32	1.09, 1.61	.005
** G3**	1.56	1.29, 1.89	<0.001	1.52	1.25, 1.85	<.001
** Unknown**	0.59	0.39, 0.88	0.010	1.03	0.67, 1.59	.900
**Procedure type**						
** Lovr-lewis**	—	—				
** Mckeown**	0.86	0.76, 0.98	0.025			
** Others**	1.39	0.93, 2.09	0.109			
**Pathologic T stage**						
** T1**	—	—		—	—	
** T2**	1.92	1.42, 2.58	<0.001	1.61	1.18, 2.22	.003
** T3**	3.18	2.44, 4.13	<0.001	2.40	1.78, 3.23	<.001
** T4**	6.12	4.46, 8.39	<0.001	3.62	2.55, 5.16	<.001
**Pathologic N stage**						
** N0**	—	—				
** N1**	2.34	2.01, 2.74	<0.001			
** N2**	3.76	3.18, 4.45	<0.001			
** N3**	4.79	3.92, 5.84	<0.001			
**Angioinvasion**						
** No/unkown**	—	—		—	—	
** Yes**	1.82	1.57, 2.11	<0.001	1.22	1.04, 1.43	.015
**Perineural invasion**						
** No/unknown**	—	—		—	—	
** Yes**	1.69	1.46, 1.95	<0.001	1.18	1.01, 1.37	.039
**Tumor size**	1.01	1.01, 1.01	<0.001	1.00	1.00, 1.01	.036
**Number of nodes examined**	1.00	1.00, 1.01	0.524			
**Number of nodes positive**	1.10	1.09, 1.11	<0.001			
**Lymph node ratio**	1.03	1.02, 1.03	<0.001	1.02	1.02, 1.02	<.001
**Treatment**						
** S group**	—	—		—	—	
** S + CT group**	0.88	0.78, 1.00	0.056	0.73	0.64, 0.83	<.001

Abbreviations: CI: confidence interval; CT: chemotherapy; HR: hazard ratio; S: surgery.

### Cut-point analysis

**Figure 1.**
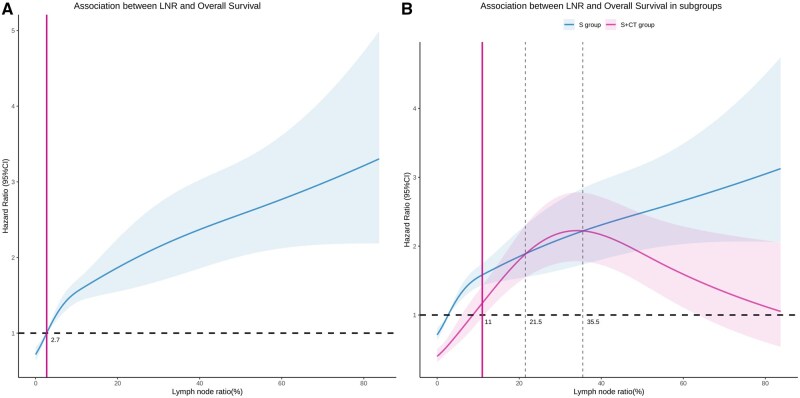
The restricted cubic splines (RCSs) plot showing the lymph node ratio (*X*-axis) and the adjusted hazard ratio of overall survival (*Y*-axis). The grey regions indicate the bounds of the 95% confidence interval (CI). Patients in the overall cohort (A) and the interaction between the lymph node ratio and treatment—surgery alone (S group) or surgery plus adjuvant chemotherapy (S + CT group) for the adjusted hazard ratio of overall survival (B). Abbreviations: S: surgery alone; S + CT: surgery plus adjuvant chemotherapy.

For a detailed evaluation, a Cox model with RCS was created to flexibly model and visualize the relationship between the LNR, treatment, and OS risk. The model was adjusted for factors that had significant effects on OS in the multivariate analysis. In the overall population, the hazard ratio (HR) for OS increased sharply with rising LNR. Specifically, the risk associated with LNR reached 1 when the LNR rose to approximately 2.7% and continued to increase thereafter (Figure 1A). The survival benefits of different treatment modalities at varying LNR levels are illustrated by RCS curves for LNR, stratified by the administration of postoperative adjuvant chemotherapy. These curves showed a consistent survival benefit for the S + CT group before the 95% CI for the LNR intersected at 11%. Beyond an LNR of 11%, the curves intersected (Figure 1B). This suggests that a survival benefit from adjuvant chemotherapy is more likely with an LNR below 11% and less likely with an LNR exceeding 11%. Therefore, we used an LNR of 11% as the threshold to distinguish the benefit of adjuvant chemotherapy after surgery.

### LNR identifies adjuvant chemotherapy benefit subpopulations

**Figure 2.**
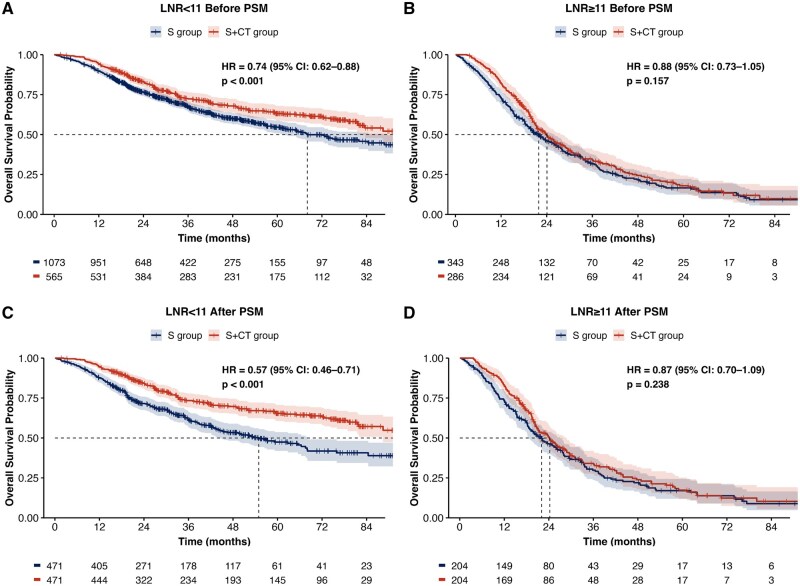
Overall survival before PSM with the treatment strategy in the LNR <11 group (A). Overall survival before PSM with the treatment strategy in the LNR ≥11 group (B) Overall survival in a matched sample with the treatment strategy in the LNR <11 group (C). Overall survival in a matched sample with the treatment strategy in the LNR ≥11 group (D). Abbreviations: LN: lymph node; LNR: lymph node ratio; S: surgery alone; PSM: propensity score matching; S + CT: surgery plus adjuvant chemotherapy.

The OS curves based on the log-rank test, stratified by LNR with a cut-off of 11%, showed that LNR ≥11% was associated with worse survival (HR: 3.12; 95% CI: 2.75-3.53; *P* < 0.001) compared to the LNR <11% group (Figure S3A). Subsequently, the overall cohort was divided into 2 categories based on LNR: <11% and ≥11%. Before PSM, subgroup analysis by LNR indicated that postoperative adjuvant chemotherapy (S + CT) conferred a significant survival benefit only in patients with LNR <11% (HR: 0.74; 95% CI: 0.62-0.88; *P* < 0.001; Figure 2A), while no significant benefit was observed in patients with LNR ≥11% (HR: 0.88; 95% CI: 0.73-1.05; *P* = 0.157; Figure 2B).

To eliminate bias between the S group and S + CT group, PSM was conducted within the LNR <11% and LNR ≥11% subgroups (Supplementary Table S2). In 942 patients with LNR <11% after PSM, the S + CT group was associated with improved survival compared to the S group (HR: 0.57; 95% CI: 0.46-0.71; *P* < 0.001; Figure 2C). In contrast, among 408 patients with LNR ≥11%, the S + CT group was not associated with improved survival compared to the S group (HR 0.87; 95% CI 0.70-1.09; *P* = 0.238; Figure 2D). To better define the subset of patients with LNR <11% who may benefit from adjuvant chemotherapy, we conducted PSM and Kaplan-Meier survival analyses in both pathologic N0 patients and those with LNR <11% but pathologic N+ disease. The results demonstrated that adjuvant chemotherapy was associated with significantly improved survival both in N0 patients (HR: 0.53; 95% CI: 0.39-0.72; *P* < 0.001; Figure S3B) and in patients with LNR <11% and pathologic N+ disease (HR: 0.67; 95% CI: 0.49-0.91; *P* = 0.0109; Figure S3C).

## Discussion

Given the poor outcomes of surgery without neoadjuvant therapy and the uncertain efficacy of adjuvant therapy, it is crucial to identify esophageal cancer subpopulations that benefit the least and the most from adjuvant chemotherapy. To the best of our knowledge, this is the first large-scale investigation in ESCC patients using a RCS method to determine LNR cut-off values. In this retrospective analysis of ESCC, we found that the effectiveness of adjuvant chemotherapy was closely linked to LNR. Specifically, adjuvant chemotherapy was associated with significantly improved survival in patients with an LNR below 11%, whereas no OS benefit was observed in those with an LNR above 11%. Our findings suggest that LNR could be a valuable tool in collaborative, interdisciplinary decision-making regarding adjuvant chemotherapy for ESCC patients.

Traditionally, postoperative adjuvant therapy for esophageal cancer has been based on the presence of positive LN (N+).^7^^,^^8^^,^^15^^,^^17^ However, some studies have shown that N0 patients can also benefit from postoperative adjuvant chemotherapy or radiotherapy.^18^^,^^19^ Even among studies focusing on pathologic N+ patients, the benefits of post-operative adjuvant chemotherapy remain controversial.^7^^,^^10^ One major reason for this discrepancy could be the inaccuracy in assessing tumor burden based on N stage. Accurate N staging depends on the number of LN removed and examined^20^^,^^21^; Moreover, even with the same *N* stage, tumor burden can vary. Theoretically, the ratio of positive LN to the total number of LN removed could serve as a valuable indicator of lymph node metastatic burden in esophageal cancer and other malignancies.

Despite the recognition of LNR’s prognostic value in various studies,^12^^,^^13^^,^^22–25^ there is no consensus on the optimal LNR threshold for guiding postoperative adjuvant chemotherapy in ESCC patients. Suggested thresholds range from 4.17% to 25%, but these discrepancies likely result from differences in sample sizes, inclusion criteria, and statistical methods.^15^^,^^22–24^^,^^26^ Our study used RCS to model and visualize the relationship between LNR, post-operative adjuvant chemotherapy, and OS, identifying an LNR of 11% as a potential threshold for benefit, not only encompassing N0 patients but also identifying potential beneficiaries among pathologic N+ patients.

Notably, when the LNR rose to around 21.5%, the mortality risk for the S + CT group temporarily exceeded that of the S group. This may be due to a higher tumor burden, where chemotherapy alone is insufficient, and more aggressive treatment is required. Detailed analyses of studies on the efficacy of postoperative adjuvant chemotherapy in pathologic N+ ESCC patients revealed that studies with positive results often had a higher proportion of pathologic N1-2 patients,^8^^,^^27^^,^^28^ while those with negative results had a higher proportion of pathologic N2-3 patients.^7^ Our study found that LNR is highly correlated with pathologic *N* stage, indicating that a higher proportion of pathologic N2-3 patients correspond to a higher LNR (Figure S2C). Conversely, the benefits of postoperative radiotherapy seem to be independent of pathologic N stage.^9^^,^^25^^,^^29^ Furthermore, some studies suggest that when LNR exceeds 23%-25%, or even below this threshold, postoperative radiotherapy still provides a survival benefit.^25^^,^^29^ Similar findings are reported in lung cancer, where LNR >50% shows significant benefits from postoperative radiotherapy.^30^ This further supports our results: patients with LNR ≤11% benefit from postoperative adjuvant chemotherapy, whereas those with higher LNR might need additional radiotherapy or chemoradiotherapy to improve survival, requiring further prospective studies for confirmation. It is noteworthy that the recent CheckMate 577 trial has provided new evidence supporting the application of immunotherapy as adjuvant treatment for esophageal cancer. This study enrolled patients who did not achieve a pathological complete response (non-pCR) after neoadjuvant chemoradiotherapy and demonstrated that adjuvant nivolumab significantly prolonged disease-free survival.^31^ Although the updated OS data presented at the 2025 ASCO annual meeting showed a favorable trend, statistical significance was not reached.^32^ Moreover, another retrospective study reported that adjuvant nivolumab following neoadjuvant DCF chemotherapy could further improve both disease-free survival and OS in patients with locally advanced ESCC, with particularly pronounced benefits observed in those with ypStage III disease.^33^ These studies suggest that patients who fail to achieve a complete pathological response may still benefit from intensified adjuvant therapy. In light of our findings, patients who undergo upfront surgery without receiving neoadjuvant therapy could theoretically be regarded as a non-pCR population, as their residual tumor burden may be comparable to that of the non-pCR patients included in the above studies. Therefore, these findings indirectly support the notion that patients undergoing primary surgery may benefit from postoperative adjuvant treatment. In particular, for subgroups with high LNR, adjuvant immunotherapy may represent a promising strategy for treatment intensification and warrants further exploration in prospective studies.

In another study on the benefits of postoperative adjuvant therapy in ESCC patients, researchers used a median LNR of 4.17% to differentiate patients who could benefit from adjuvant therapy.^15^ They found that patients with fewer than 28 LN removed and an LNR >4.17% benefited from postoperative adjuvant therapy (*P* = 0.030). This threshold included patients with LNRs between 4.17% and 11%. Since the study did not distinguish between adjuvant chemotherapy and radiotherapy, it showed that patients with an LNR >11% also benefited from adjuvant therapy. Additionally, even patients with an LNR <4.17% showed a trend of benefit from postoperative adjuvant therapy, though not statistically significant. Interestingly, another study using RCS curves to determine the benefits of postoperative adjuvant chemotherapy in esophageal adenocarcinoma (EAC) patients set an LNR >12.3% as the threshold for adjuvant chemotherapy benefit,^24^ seemingly contradicting our results. Upon closer examination, the differences likely stem from variations in pathological types and study design. First, their study focused on EAC patients, whose treatment responses may differ from those of ESCC patients, as reflected in the CROSS study, which showed greater benefits for ESCC patients undergoing neoadjuvant chemoradiotherapy.^1^ Second, their median number of LN removed was only 14, compared to 20 in our study, suggesting their LNR might be overestimated due to insufficient lymph node removal, leading to observed survival benefits in patients with higher LNR. Notably, a similar phenomenon was observed in our study: when the LNR exceeded 35.5%, the RCS curve for the S + CT group began to decline. Further analysis revealed that in patients with LNR >35.5%, those with fewer than 15 LN removed still benefited from postoperative adjuvant chemotherapy (Figure S3D-E). This suggests that the high LNR in these patients might be overestimated due to insufficient lymph node removal. Therefore, these patients are not truly high-LNR patients and can still benefit from postoperative adjuvant chemotherapy. Our findings suggest that while an LNR <11% is associated with improved survival following adjuvant chemotherapy, the benefit may extend to select patients with LNR >11%, especially in the context of limited nodal evaluation. Therefore, the LNR should be interpreted in conjunction with other clinical and pathological factors.

However, our study does have certain limitations. First, since the data were gathered retrospectively, it is difficult to draw firm conclusions on whether the LNR or AJCC pathologic N categories are better for identifying patients with resectable ESCC who may benefit most from adjuvant treatment. Second, our results are based on a single institutional study that included 2267 esophagectomy patients in China with ESCC, whether our results and cut-off LNR values can be applied to other institutions remains to be demonstrated. Additionally, although we adjusted for several clinicopathologic variables, potential confounding cannot be fully ruled out. Some patients who did not receive adjuvant chemotherapy may have had poor functional status or comorbidities that made them ineligible for further treatment, which could have negatively affected their survival regardless of treatment. However, detailed information on performance status or specific reasons for not receiving adjuvant therapy was not consistently available in our dataset.

## Conclusion

In conclusion, LNR is an important prognostic indicator that can help identify ESCC patients who may benefit from postoperative adjuvant chemotherapy. For patients with higher LNRs, more intensive treatments such as postoperative radiotherapy or chemoradiotherapy may be necessary to improve survival. However, these findings warrant further large-scale prospective studies for validation.

## Supplementary Material

oyaf315_Supplementary_Data

## Data Availability

Further details and other data supporting the results of this study are available from the corresponding author upon request.
