# Supplementary material for: Lymph node ratio predicts adjuvant chemotherapy benefit in esophageal squamous cell carcinoma
Source: Oncologist. 2025 Sep 25;30(10):oyaf315. doi: 10.1093/oncolo/oyaf315 (PMC12527439; doi:10.1093/oncolo/oyaf315)
Supplement: oyaf315_Supplementary_Data [file oyaf315_supplementary_data.zip › Supplemental figure captions.docx]

**Supplemental figure captions**

Supplementary Figure 1. Diagram of patient selection. CT: chemotherapy; RT, radiotherapy; CRT: chemoradiotherapy.

Supplementary Figure 2. Spearman’s correlation analysis showing significant correlations between lymph node ratios and the number of nodes examined (A), the number of positive nodes (B), and the pathologic N stage (C), with particularly strong correlations for the number of positive nodes (R = 0.970, p-value < 0.001) and the pathologic N stage (R = 0.964, p-value < 0.001).

Supplementary Figure 3. Overall survival of patients with a lymph node ratio <11 or ≥11 in the whole group (A). Overall survival in a matched sample with the treatment strategy in the lymph node ratio <11 and N0 group (B). Overall survival in a matched sample with the treatment strategy in the lymph node ratio <11 and N+ group (C). Overall survival of patients with lymph node ratio ≥35.5% and <15 lymph nodes examined (LNE) according to treatment strategy (D). Overall survival of patients with lymph node ratio ≥35.5% and ≥15 lymph nodes examined (LNE) according to treatment strategy (E). LNR: lymph node ratio; LNE: lymph node examined; S: surgery alone; S+CT: surgery plus adjuvant chemotherapy.
