# Supplementary material for: Lymph node ratio predicts adjuvant chemotherapy benefit in esophageal squamous cell carcinoma
Source: Oncologist. 2025 Sep 25;30(10):oyaf315. doi: 10.1093/oncolo/oyaf315 (PMC12527439; doi:10.1093/oncolo/oyaf315)
Supplement: oyaf315_Supplementary_Data [file oyaf315_supplementary_data.zip › Supplementary Table.docx]

**Supplementary Table1 S**ummarizes chemotherapy regimens for the S+CT group

| chemotherapy regimens | n | % |
| --- | --- | --- |
| *Nedaplatin-based* | 342 | 40.2% |
| Nedaplatin and Fluorouracil | 158 | 18.6% |
| Nedaplatin and Paclitaxel | 146 | 17.2% |
| Nedaplatin and Gemcitabine | 24 | 2.8% |
| Nedaplatin | 13 | 1.5% |
| Nedaplatin and Etoposide | 1 | 0.1% |
| *Cisplatin-based* | 247 | 29.0% |
| Cisplatin and Paclitaxel | 184 | 21.6% |
| Cisplatin and Fluorouracil | 59 | 6.9% |
| Cisplatin | 3 | 0.4% |
| Cisplatin and Etoposide | 1 | 0.1% |
| *Oxaliplatin-based* | 202 | 23.7% |
| Oxaliplatin and Fluorouracil | 125 | 14.7% |
| Oxaliplatin and Paclitaxel | 53 | 6.2% |
| Oxaliplatin and Gemcitabine | 16 | 1.9% |
| Oxaliplatin and Irinotecan | 7 | 0.8% |
| Oxaliplatin | 1 | 0.1% |
| *Carboplatin-based* | 15 | 1.8% |
| Carboplatin and Paclitaxel | 15 | 1.8% |
| *Capecitabine or S1* | 45 | 5.3% |
| Total | 851 | 100.0% |

Note: S1: Gimeracil and Oteracil Potassium Capsules

Supplementary Table2. Clinical characteristics in patients with sub-groups before and after 1:1 propensity matching

|  | Before propensity matching | | |  | After propensity matching | | |
| --- | --- | --- | --- | --- | --- | --- | --- |
|  | S group | S+CT group | p-value |  | S group | S+CT group | p-value |
| *LNR<11* |  |  |  |  |  |  |  |
| **Number of patients** | 1,073 | 565 |  |  | 471 | 471 |  |
| **Sex** |  |  | 0.073 |  |  |  | 0.613 |
| female | 232 (22%) | 101 (18%) |  |  | 83 (18%) | 89 (19%) |  |
| male | 841 (78%) | 464 (82%) |  |  | 388 (82%) | 382 (81%) |  |
| **Age (median [IQR])** | 64 (59, 70) | 60 (54, 64) | <0.001 |  | 61 (56, 66) | 61 (56, 65) | 0.629 |
| **Tumor location** |  |  | <0.001 |  |  |  | 0.853 |
| upper third | 295 (27%) | 106 (19%) |  |  | 111 (24%) | 104 (22%) |  |
| middle third | 590 (55%) | 299 (53%) |  |  | 251 (53%) | 254 (54%) |  |
| lower third | 188 (18%) | 160 (28%) |  |  | 109 (23%) | 113 (24%) |  |
| **Tumor grade** |  |  | 0.005 |  |  |  | 0.771 |
| G1 | 198 (18%) | 107 (19%) |  |  | 92 (20%) | 88 (19%) |  |
| G2 | 422 (39%) | 222 (39%) |  |  | 189 (40%) | 191 (41%) |  |
| G3 | 378 (35%) | 220 (39%) |  |  | 180 (38%) | 177 (38%) |  |
| unknown | 75 (7.0%) | 16 (2.8%) |  |  | 10 (2.1%) | 15 (3.2%) |  |
| **Procedure type** |  |  | <0.001 |  |  |  | 0.779 |
| Ivor–Lewis | 176 (16%) | 232 (41%) |  |  | 143 (30%) | 152 (32%) |  |
| Mckeown | 884 (82%) | 324 (57%) |  |  | 321 (68%) | 311 (66%) |  |
| Others | 13 (1.2%) | 9 (1.6%) |  |  | 7 (1.5%) | 8 (1.7%) |  |
| **Pathologic T stage** |  |  | <0.001 |  |  |  | 0.778 |
| T1 | 221 (21%) | 39 (6.9%) |  |  | 30 (6.4%) | 38 (8.1%) |  |
| T2 | 240 (22%) | 112 (20%) |  |  | 99 (21%) | 97 (21%) |  |
| T3 | 550 (51%) | 397 (70%) |  |  | 323 (69%) | 319 (68%) |  |
| T4 | 62 (5.8%) | 17 (3.0%) |  |  | 19 (4.0%) | 17 (3.6%) |  |
| **Pathologic N stage** |  |  | <0.001 |  |  |  | 0.396 |
| N0 | 757 (71%) | 327 (58%) |  |  | 274 (58%) | 289 (61%) |  |
| N1 | 281 (26%) | 209 (37%) |  |  | 169 (36%) | 162 (34%) |  |
| N2 | 35 (3.3%) | 28 (5.0%) |  |  | 28 (5.9%) | 20 (4.2%) |  |
| N3 | 0 (0%) | 1 (0.2%) |  |  | 0 (0%) | 0 (0%) |  |
| **Angioinvasion** |  |  | <0.001 |  |  |  | 0.847 |
| No/Unkown | 980 (91%) | 484 (86%) |  |  | 410 (87%) | 408 (87%) |  |
| Yes | 93 (8.7%) | 81 (14%) |  |  | 61 (13%) | 63 (13%) |  |
| **Perineural invasion** |  |  | 0.776 |  |  |  | 0.792 |
| No/Unkown | 902 (84%) | 478 (85%) |  |  | 392 (83%) | 395 (84%) |  |
| Yes | 171 (16%) | 87 (15%) |  |  | 79 (17%) | 76 (16%) |  |
| **Tumor size mm (median [IQR])** | 35 (25, 50) | 40 (28, 50) | 0.008 |  | 35 (25, 50) | 40 (27, 50) | 0.488 |
| **Number of nodes examined (median [IQR])** | 20 (13, 28) | 20 (14, 28) | 0.104 |  | 22 (14, 30) | 20 (14, 28) | 0.160 |
| **Number of nodes positive (median [IQR])** | 0.00 (0.00, 1.00) | 0.00 (0.00, 1.00) | <0.001 |  | 0.00 (0.00, 1.00) | 0.00 (0.00, 1.00) | 0.228 |
| **Lymph node ratio (median [IQR])** | 0.00 (0.00, 3.45) | 0.00 (0.00, 5.56) | <0.001 |  | 0.00 (0.00, 4.88) | 0.00 (0.00, 4.88) | 0.408 |
| *LNR≥11* |  |  |  |  |  |  |  |
| **Number of patients** | 343 | 286 |  |  | 204 | 204 |  |
| **Sex** |  |  | 0.176 |  |  |  | 0.673 |
| female | 59 (17%) | 38 (13%) |  |  | 28 (14%) | 31 (15%) |  |
| male | 284 (83%) | 248 (87%) |  |  | 176 (86%) | 173 (85%) |  |
| **Age (median [IQR])** | 64 (58, 70) | 60 (53, 65) | <0.001 |  | 61 (55, 67) | 61 (55, 66) | 0.895 |
| **Tumor location** |  |  | <0.001 |  |  |  | 0.667 |
| upper third | 93 (27%) | 38 (13%) |  |  | 38 (19%) | 35 (17%) |  |
| middle third | 181 (53%) | 149 (52%) |  |  | 108 (53%) | 117 (57%) |  |
| lower third | 69 (20%) | 99 (35%) |  |  | 58 (28%) | 52 (25%) |  |
| **Tumor grade** |  |  | 0.694 |  |  |  | 0.836 |
| G1 | 39 (11%) | 28 (9.8%) |  |  | 26 (13%) | 22 (11%) |  |
| G2 | 148 (43%) | 130 (45%) |  |  | 90 (44%) | 87 (43%) |  |
| G3 | 149 (43%) | 125 (44%) |  |  | 84 (41%) | 92 (45%) |  |
| unknown | 7 (2.0%) | 3 (1.0%) |  |  | 4 (2.0%) | 3 (1.5%) |  |
| **Procedure type** |  |  | <0.001 |  |  |  | 0.951 |
| Ivor–Lewis | 81 (24%) | 137 (48%) |  |  | 75 (37%) | 74 (36%) |  |
| Mckeown | 253 (74%) | 139 (49%) |  |  | 122 (60%) | 124 (61%) |  |
| Others | 9 (2.6%) | 10 (3.5%) |  |  | 7 (3.4%) | 6 (2.9%) |  |
| **Pathologic T stage** |  |  | 0.617 |  |  |  | 0.993 |
| T1 | 10 (2.9%) | 9 (3.1%) |  |  | 7 (3.4%) | 8 (3.9%) |  |
| T2 | 58 (17%) | 49 (17%) |  |  | 37 (18%) | 37 (18%) |  |
| T3 | 227 (66%) | 198 (69%) |  |  | 136 (67%) | 136 (67%) |  |
| T4 | 48 (14%) | 30 (10%) |  |  | 24 (12%) | 23 (11%) |  |
| **Pathologic N stage** |  |  | 0.556 |  |  |  | 0.525 |
| N0 | 0 (0%) | 0 (0%) |  |  | 0 (0%) | 0 (0%) |  |
| N1 | 83 (24%) | 61 (21%) |  |  | 46 (23%) | 46 (23%) |  |
| N2 | 166 (48%) | 137 (48%) |  |  | 88 (43%) | 98 (48%) |  |
| N3 | 94 (27%) | 88 (31%) |  |  | 70 (34%) | 60 (29%) |  |
| **Angioinvasion** |  |  | 0.345 |  |  |  | 0.243 |
| No/Unkown | 229 (67%) | 201 (70%) |  |  | 133 (65%) | 144 (71%) |  |
| Yes | 114 (33%) | 85 (30%) |  |  | 71 (35%) | 60 (29%) |  |
| **Perineural invasion** |  |  | 0.868 |  |  |  | 0.409 |
| No/Unkown | 261 (76%) | 216 (76%) |  |  | 154 (75%) | 161 (79%) |  |
| Yes | 82 (24%) | 70 (24%) |  |  | 50 (25%) | 43 (21%) |  |
| **Tumor size mm (median [IQR])** | 40 (30, 60) | 40 (30, 50) | 0.221 |  | 40 (30, 55) | 40 (30, 51) | 0.818 |
| **Number of nodes examined (median [IQR])** | 19 (13, 27) | 19 (13, 26) | 0.768 |  | 19 (13, 27) | 19 (13, 26) | 0.705 |
| **Number of nodes positive (median [IQR])** | 4.00 (3.00, 7.00) | 4.00 (3.00, 7.00) | 0.783 |  | 5.00 (3.00, 8.00) | 4.00 (3.00, 7.00) | 0.329 |
| **Lymph node ratio (median [IQR])** | 21 (14, 33) | 24 (16, 36) | 0.193 |  | 23 (15, 37) | 23 (15, 36) | 0.667 |
| *LNR<11 and N0* |  |  |  |  |  |  |  |
| **Number of patients** | 757 | 327 |  |  | 266 | 266 |  |
| **Sex** |  |  | 0.117 |  |  |  | >0.999 |
| Female | 176 (23%) | 62 (19%) |  |  | 53 (20%) | 53 (20%) |  |
| Male | 581 (77%) | 265 (81%) |  |  | 213 (80%) | 213 (80%) |  |
| **Age (median [IQR])** | 64 (59, 70) | 60 (55, 64) | <0.001 |  | 61 (56, 66) | 61 (57, 65) | 0.747 |
| **Tumor location** |  |  | <0.001 |  |  |  | 0.907 |
| upper third | 201 (27%) | 64 (20%) |  |  | 61 (23%) | 58 (22%) |  |
| middle third | 427 (56%) | 176 (54%) |  |  | 143 (54%) | 148 (56%) |  |
| lower third | 129 (17%) | 87 (27%) |  |  | 62 (23%) | 60 (23%) |  |
| **Tumor grade** |  |  | 0.002 |  |  |  | 0.482 |
| G1 | 149 (20%) | 75 (23%) |  |  | 69 (26%) | 62 (23%) |  |
| G2 | 289 (38%) | 131 (40%) |  |  | 98 (37%) | 108 (41%) |  |
| G3 | 253 (33%) | 113 (35%) |  |  | 95 (36%) | 88 (33%) |  |
| Unknown | 66 (8.7%) | 8 (2.4%) |  |  | 4 (1.5%) | 8 (3.0%) |  |
| **Procedure type** |  |  | <0.001 |  |  |  | 0.889 |
| Ivor–Lewis | 128 (17%) | 143 (44%) |  |  | 88 (33%) | 94 (35%) |  |
| Mckeown | 620 (82%) | 178 (54%) |  |  | 175 (66%) | 169 (64%) |  |
| Others | 9 (1.2%) | 6 (1.8%) |  |  | 3 (1.1%) | 3 (1.1%) |  |
| **Pathologic T stage** |  |  | <0.001 |  |  |  | 0.274 |
| T1 | 195 (26%) | 24 (7.3%) |  |  | 13 (4.9%) | 23 (8.6%) |  |
| T2 | 177 (23%) | 63 (19%) |  |  | 58 (22%) | 55 (21%) |  |
| T3 | 349 (46%) | 233 (71%) |  |  | 185 (70%) | 182 (68%) |  |
| T4 | 36 (4.8%) | 7 (2.1%) |  |  | 10 (3.8%) | 6 (2.3%) |  |
| **Angioinvasion** |  |  | 0.014 |  |  |  | 0.653 |
| No/Unkown | 714 (94%) | 295 (90%) |  |  | 240 (90%) | 243 (91%) |  |
| Yes | 43 (5.7%) | 32 (9.8%) |  |  | 26 (9.8%) | 23 (8.6%) |  |
| **Perineural invasion** |  |  | 0.807 |  |  |  | 0.533 |
| No/Unkown | 657 (87%) | 282 (86%) |  |  | 226 (85%) | 231 (87%) |  |
| Yes | 100 (13%) | 45 (14%) |  |  | 40 (15%) | 35 (13%) |  |
| **Tumor size mm**  **(median [IQR])** | 30 (20, 50) | 38 (26, 47) | 0.003 |  | 37 (30, 50) | 39 (25, 50) | 0.497 |
| **Number of nodes examined (median [IQR])** | 17 (12, 24) | 18 (13, 25) | 0.387 |  | 18 (12, 26) | 18 (12, 25) | 0.975 |
| *LNR<11 and N+* |  |  |  |  |  |  |  |
| **Number of patients** | 316 | 238 |  |  | 179 | 179 |  |
| **Sex** |  |  | 0.680 |  |  |  | >0.999 |
| female | 56 (18%) | 39 (16%) |  |  | 32 (18%) | 32 (18%) |  |
| male | 260 (82%) | 199 (84%) |  |  | 147 (82%) | 147 (82%) |  |
| **Age (median [IQR])** | 64 (58, 69) | 60 (54, 64) | <0.001 |  | 60 (56, 66) | 62 (57, 65) | 0.350 |
| **Tumor location** |  |  | <0.001 |  |  |  | 0.546 |
| upper third | 94 (30%) | 42 (18%) |  |  | 46 (26%) | 38 (21%) |  |
| middle third | 163 (52%) | 123 (52%) |  |  | 94 (53%) | 96 (54%) |  |
| lower third | 59 (19%) | 73 (31%) |  |  | 39 (22%) | 45 (25%) |  |
| **Tumor grade** |  |  | 0.582 |  |  |  | 0.959 |
| G1 | 49 (16%) | 32 (13%) |  |  | 22 (12%) | 25 (14%) |  |
| G2 | 133 (42%) | 91 (38%) |  |  | 76 (42%) | 72 (40%) |  |
| G3 | 125 (40%) | 107 (45%) |  |  | 74 (41%) | 75 (42%) |  |
| unknown | 9 (2.8%) | 8 (3.4%) |  |  | 7 (3.9%) | 7 (3.9%) |  |
| **Procedure type** |  |  | <0.001 |  |  |  | 0.855 |
| Ivor–Lewis | 48 (15%) | 89 (37%) |  |  | 41 (23%) | 47 (26%) |  |
| Mckeown | 264 (84%) | 146 (61%) |  |  | 135 (75%) | 129 (72%) |  |
| Others | 4 (1.3%) | 3 (1.3%) |  |  | 3 (1.7%) | 3 (1.7%) |  |
| **Pathologic T stage** |  |  | 0.197 |  |  |  | 0.873 |
| T1 | 26 (8.2%) | 15 (6.3%) |  |  | 16 (8.9%) | 12 (6.7%) |  |
| T2 | 63 (20%) | 49 (21%) |  |  | 33 (18%) | 34 (19%) |  |
| T3 | 201 (64%) | 164 (69%) |  |  | 119 (66%) | 123 (69%) |  |
| T4 | 26 (8.2%) | 10 (4.2%) |  |  | 11 (6.1%) | 10 (5.6%) |  |
| **Pathologic N stage** |  |  | 0.579 |  |  |  | >0.999 |
| N1 | 281 (89%) | 209 (88%) |  |  | 156 (87%) | 157 (88%) |  |
| N2 | 35 (11%) | 28 (12%) |  |  | 23 (13%) | 22 (12%) |  |
| N3 | 0 (0%) | 1 (0.4%) |  |  | 0 (0%) | 0 (0%) |  |
| **Angioinvasion** |  |  | 0.147 |  |  |  | 0.893 |
| No/Unkown | 266 (84%) | 189 (79%) |  |  | 144 (80%) | 145 (81%) |  |
| Yes | 50 (16%) | 49 (21%) |  |  | 35 (20%) | 34 (19%) |  |
| **Perineural invasion** |  |  | 0.163 |  |  |  | 0.114 |
| No/Unkown | 245 (78%) | 196 (82%) |  |  | 137 (77%) | 149 (83%) |  |
| Yes | 71 (22%) | 42 (18%) |  |  | 42 (23%) | 30 (17%) |  |
| **Tumor size mm (median [IQR])** | 40 (30, 54) | 40 (30, 50) | 0.461 |  | 35 (28, 50) | 40 (30, 50) | 0.139 |
| **Number of nodes examined (median [IQR])** | 25 (19, 34) | 24 (17, 31) | 0.321 |  | 27 (19, 35) | 24 (18, 32) | 0.130 |
| **Number of nodes positive (median [IQR])** | 1.00 (1.00, 2.00) | 1.00 (1.00, 2.00) | 0.9046 |  | 1.00 (1.00, 2.00) | 1.00 (1.00, 2.00) | 0.6647 |
| **Lymph node ratio (median [IQR])** | 5.88 (4.00, 8.33) | 6.25 (4.35, 7.69) | 0.526 |  | 5.71 (3.92, 7.95) | 5.88 (4.17, 7.69) | 0.363 |
| Values are mean ± standard deviation or number (percentage).  LNR: Lymph node ratio; S: surgery; CT: chemotherapy. | | | | | | | |
