# Supplementary figures and images for: Lymph node ratio predicts adjuvant chemotherapy benefit in esophageal squamous cell carcinoma
Source: Oncologist. 2025 Sep 25;30(10):oyaf315. doi: 10.1093/oncolo/oyaf315 (PMC12527439; doi:10.1093/oncolo/oyaf315)

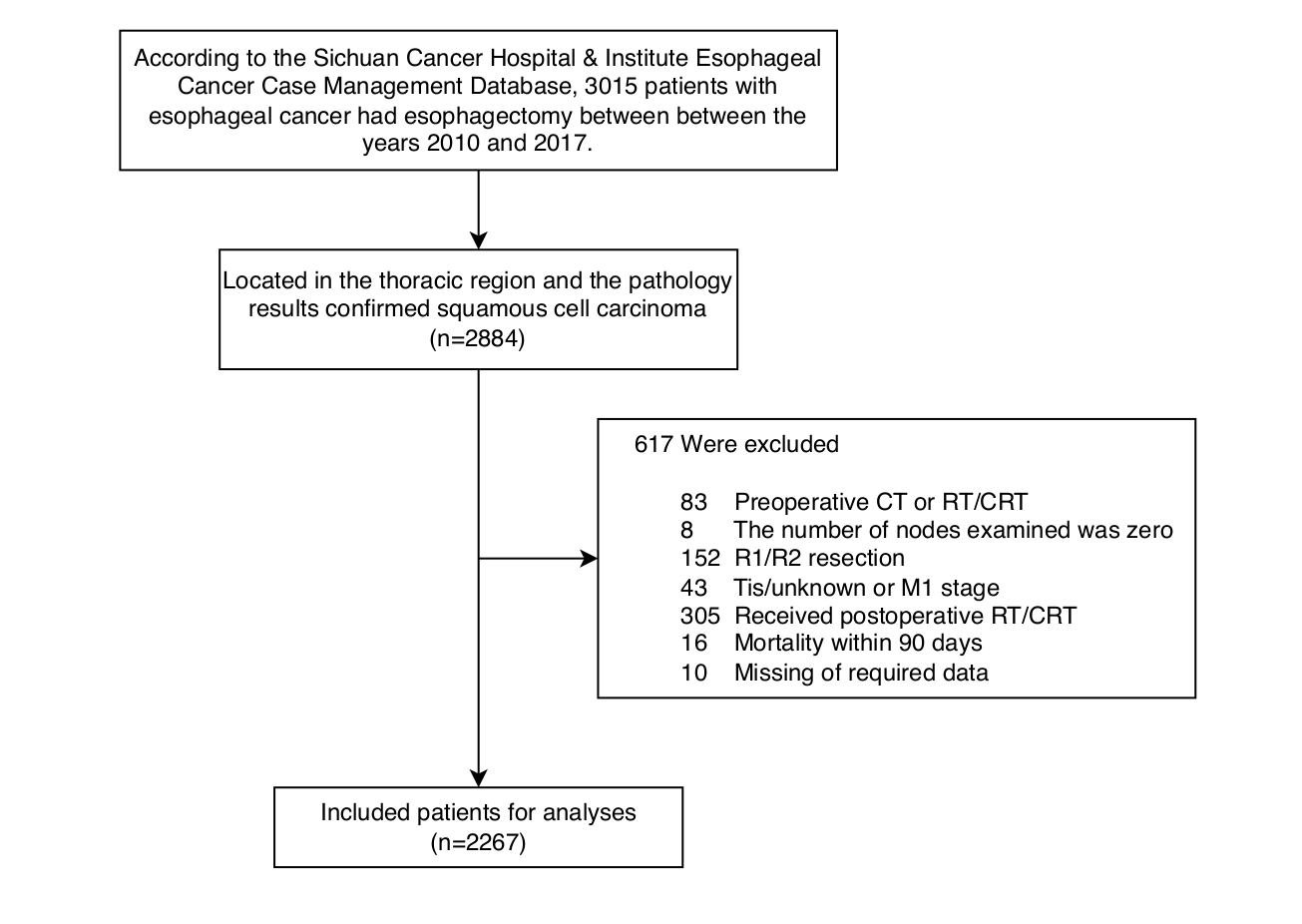

Supplement: oyaf315_Supplementary_Data [file oyaf315_supplementary_data.zip › Supplementary Figure 1.tif]

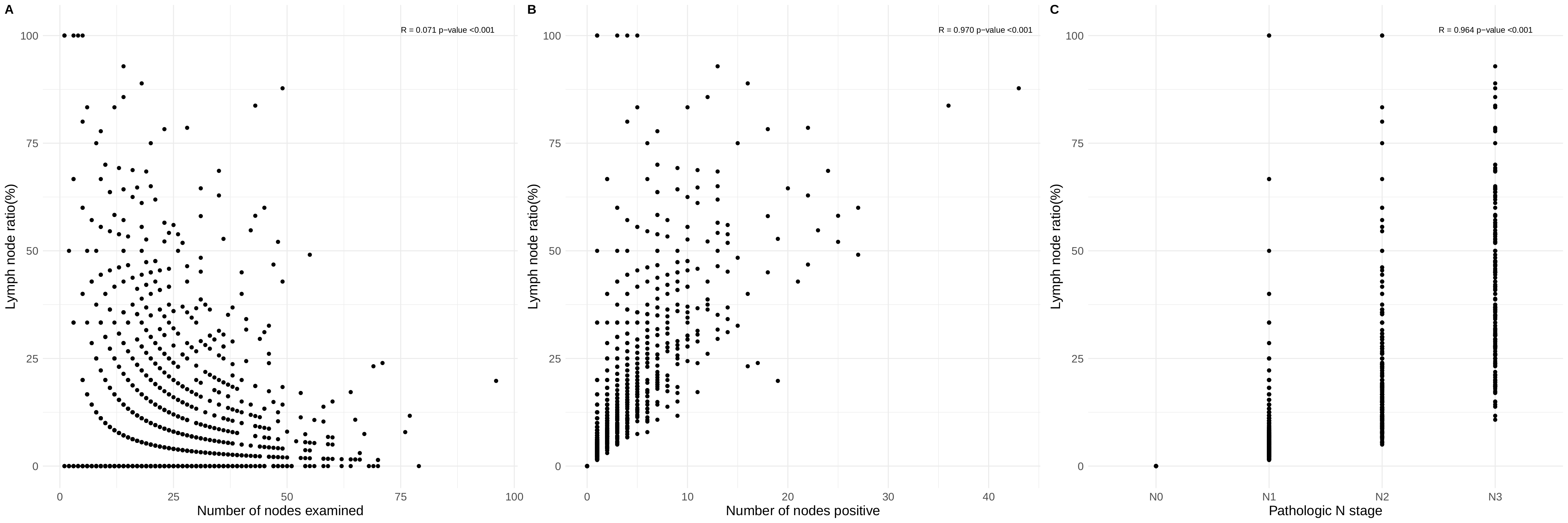

Supplement: oyaf315_Supplementary_Data [file oyaf315_supplementary_data.zip › Supplementary Figure 2.tif]

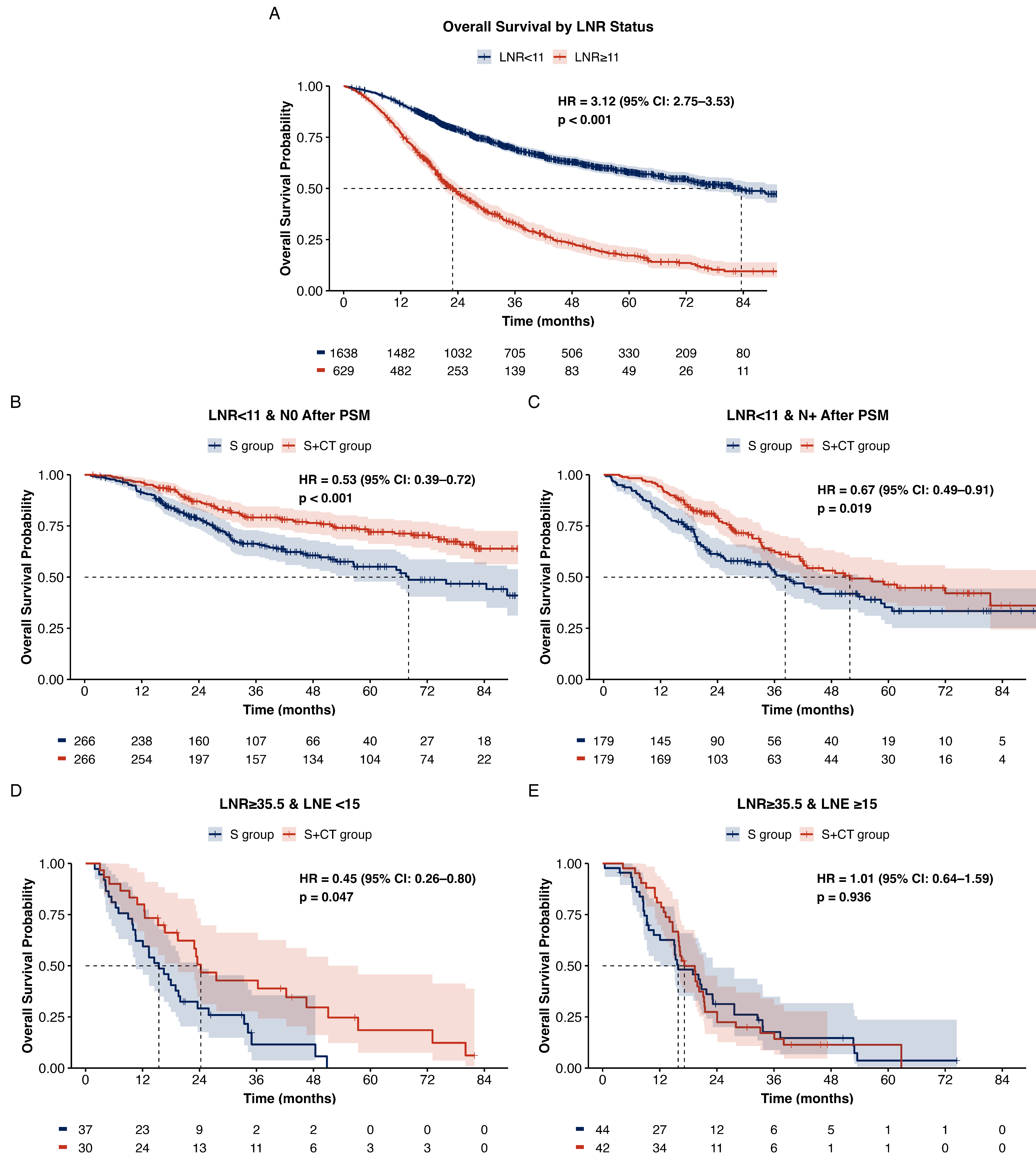

Supplement: oyaf315_Supplementary_Data [file oyaf315_supplementary_data.zip › Supplementary Figure 3.tif]
